# Time to Death by Suicide in an Epidemiological Sample of Veterans With an Inpatient Hospitalization for Heart Failure

**DOI:** 10.1016/j.jagp.2025.02.005

**Published:** 2025-02-18

**Authors:** Melanie L. Bozzay, Matthew F. Thompson, Lan Jiang, Jennifer M. Primack, John E. McGeary, Alyssa N. De Vito, Julia Browne, Catherine M. Kelso, James L. Rudolph, Zachary J. Kunicki

**Affiliations:** From the Wexner Medical Center (MLB), The Ohio State University, Columbus OH; Butler Hospital (ANDV), Providence, RI; VA Center of Innovation in Long Term Services (LJ, JMP, JEMG, JB, JLR, ZJK), Providence VA Medical Center, Providence, RI; Department of Psychiatry & Human Behavior (MFT, JMP, JEMG, ANDV, JB, ZJK), Alpert Medical School of Brown University, Providence, RI; Department of Medicine (JLR), Alpert Medical School of Brown University, Providence, RI; VA RR&D Center for Neurorestoration and Neurotechnology (CMK), Providence VA Medical Center, Providence, RI; and the Veterans Health Administration (CMK), Office of Patient Care Services, Geriatrics and Extended Care, Washington, DC. Send correspondence and reprint requests to Melanie L. Bozzay, Ph.D., The Ohio State University, 1960 Kenny Rd, Columbus OH, 43210.

**Keywords:** Suicide, heart failure, Veteran, Mortality, post-discharge

## Abstract

**Background:**

Patients who have experienced an inpatient hospitalization for heart failure are at increased risk of mortality, particularly during the months following discharge. This study described patient characteristics associated with suicide death and examined the time course of death by suicide compared to that of other types of death amongst patients with a recent medical hospitalization for heart failure.

**Method:**

Using Department of Veterans Affairs (VA) electronic medical records from 2011 to 2020, we identified a cohort of Veterans hospitalized with a heart failure diagnosis who died after discharge. We merged the VA Mortality Database Record, a compilation of death sources and causes, with the VA electronic health record and compared characteristics of Veterans who died by suicide and by other causes.

**Results:**

In the cohort of 348,840 Veterans, 1,097 died by suicide and 347,743 died by other causes. Compared to those who died by other causes, Veterans who died by suicide were, on average, younger, had fewer comorbidities, more likely to have a depression diagnosis, more likely to be White, and had lower prior year healthcare costs (Standardized mean differences [SMD] ranged from 0.25 to 0.46). Unadjusted analyses showed longer length of time between hospital discharge and death for those who died by suicide compared to other causes (SMD = 0.18); however, analyses adjusting for comorbidities revealed no difference in time to death between those who died by suicide versus other causes.

**Conclusions:**

Demographic, clinical, and healthcare utilization characteristics distinguished Veterans with heart failure who died by suicide from those who died by other causes. Time to death following hospital discharge did not differ between groups when accounting for relevant factors. Comprehensive suicide screening and intervention is needed following a heart-failure hospital discharge, particularly for Veterans at elevated risk.

## SUMMARY

In a large sample, this study found that patients with HF who died by suicide were younger, more likely to be White, and had fewer medical comorbidities than those who died by other causes. Those who died by suicide had a longer time to death, a finding which was ameliorated after accounting for relevant demographics, medical comorbidities, and healthcare utilization. These findings suggest there may be missed opportunities for clinical intervention among HF patients in the transition from medical hospitalization.

## OBJECTIVE

Heart failure (HF) is highly prevalent in the United States and worldwide.^1^ It is a leading cause of inpatient admissions,^2^ healthcare expenditures, and premature death.^1^ Unfortunately, patients who have experienced an inpatient hospitalization for HF are at increased risk of mortality, particularly during the months following discharge.^3^ This increased risk may be because HF is associated with significant morbidity and functional challenges that can complicate disease progression and impair functioning, factors that are often more severe in recently hospitalized patients, and that can increase risk of death^4,5^ in general. Identifying modifiable factors that could reduce mortality or prolong the life course among patients with HF is thus an important objective of research. As suicide is behaviorally driven, and thus to some extent, preventable, this study focuses on 1) identifying factors that distinguish patients with HF who die by suicide versus other forms of death and 2) examining the time course of death among patients who died by suicide versus other means.

Patients with a recent HF admission may be vulnerable to dying by suicide, especially in the months following discharge. Research suggests that patients with particular psychiatric presentations (i.e., depression, substance abuse) that have been implicated in suicide risk^6,7^ are more likely to develop coronary disease, experience a worse symptom course, and require inpatient medical hospitalization.^8-12^ As such, it is possible that an important subset of patients hospitalized for HF are vulnerable to dying by suicide. Moreover, HF has a high burden of disease and is often linked with comorbid medical conditions, factors that can contribute to severe distress, reflected in difficulties in psychosocial and daily functioning,^13^ deficits in quality of life,^14^ and psychiatric concerns (i.e., depression),^15^ in turn increasing the risk of suicide.^16^ Indeed, studies implicate inpatient admission for HF in increased risk of suicide.^17,18^ However, research is critically needed to more comprehensively identify whether these demographic, psychiatric, or medical characteristics demarcate the subset of patients with a recent HF admission who die by suicide.

It is also possible that the time course of death varies among patients with a recent HF admission who die by suicide versus other causes. Some epidemiological studies comparing patients diagnosed with HF to matched controls have found elevated suicide risk in the period of time immediately following a HF diagnosis, with risk being most elevated 3 to and 6 months post HF diagnosis, and declining thereafter.^17,19^ These findings suggest that there are periods of time that are particularly risky for suicide for individuals with HF. The post-discharge period may be a particularly risky period of time for these individuals, as patients face significant challenges in the period of time following medical hospitalization, including adjusting to new medical regimens and life routines, and potentially reduced physical function, in addition to managing other life stressors.^20,21^ These factors could increase the risk of suicide in comparison to other forms of death, especially in the immediate months following discharge from a medical inpatient stay. However, they could also contribute to premature death by other causes. However, whether risk for suicide is elevated in the period shortly following a medical hospitalization for HF is unclear, and how the time course of death by suicide compares to that of other types of death amongst HF patients is unknown. These research questions have high relevance to efforts to improve outcomes among HF patients, as suicide deaths are behaviorally driven, and thus to some extent, preventable.

This study sought to address these critical knowledge gaps via two primary aims. First, we identified characteristics that differentiated patients with a history of a HF-related inpatient stay who died by suicide versus all other forms of death in a large sample. Second, we examined whether the time to death after discharge from a medical inpatient stay differed amongst patients who died by suicide. We expected that the time to death would be shorter for patients who died by suicide versus other forms of death.

## METHODS

### Participants and Procedures

**FIGURE 1.**
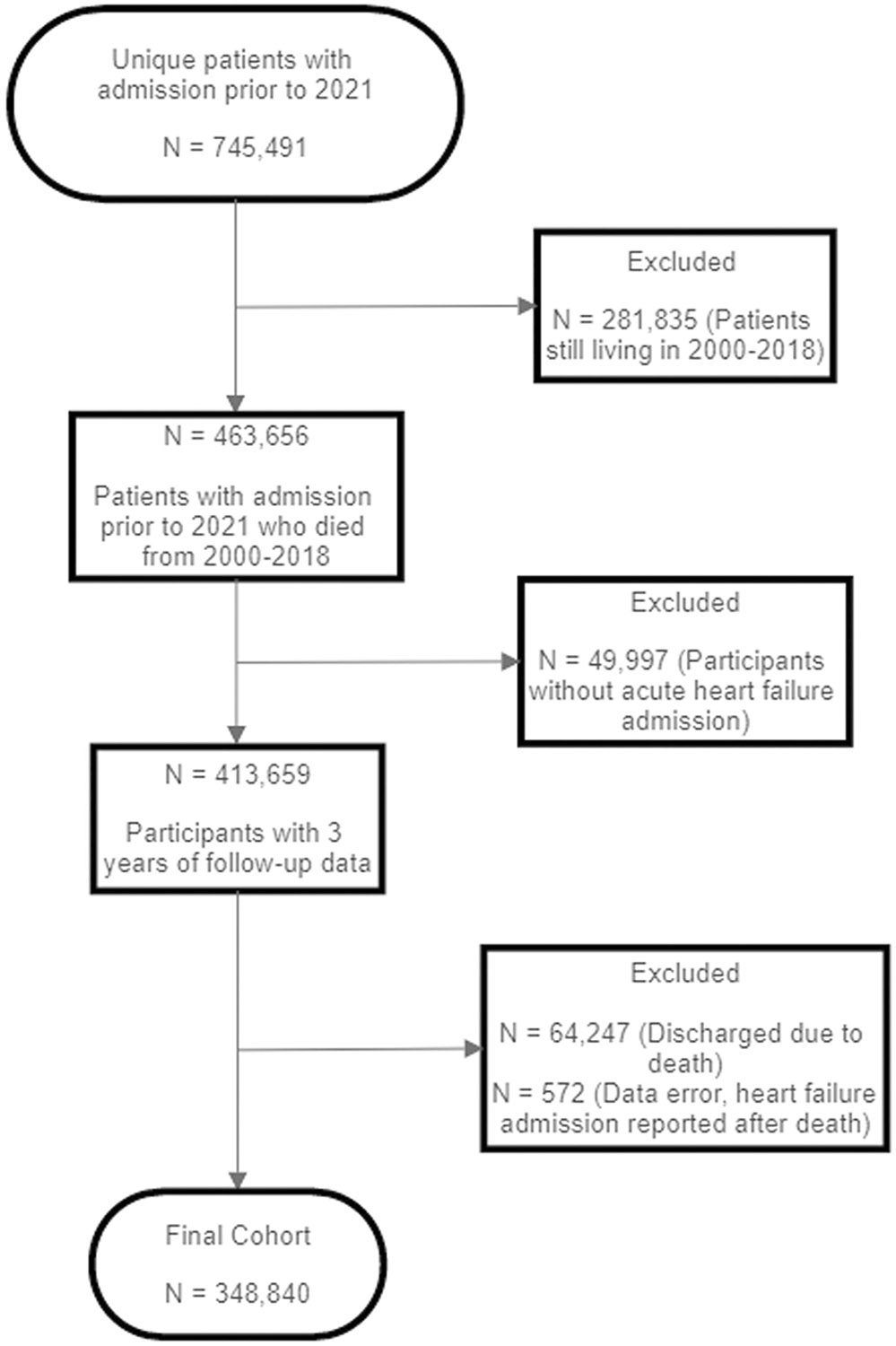
Study flowchart.

This was a retrospective cohort study derived from a national population of 745,491 Veterans with a primary admission diagnosis of heart failure who visited any of 129 different Department of Veterans Affairs (VA) Medical Centers between October 1, 2011, and September 30, 2020. We included patients with at least one heart failure-related hospital admission. In cases where patients had multiple hospital admissions, we selected the last admission before their death. We excluded participants who were still living between 2000 and 2018, who did not have an acute heart failure admission for their most recent hospital admission prior to death, were discharged due to death, and who did not have 3 years of follow-up data. Our final cohort was 348,840 unique Veterans. See Fig. 1 for the study flow chart. VA electronic records were used to collect information about demographics, comorbidities, and prior health care utilization. These data were merged with mortality data from the VA Mortality Data Repository (MDR), which integrates information from the National Death Index and VA administrative source to form the most comprehensive database available about Veteran mortality.^22^ All study procedures were approved by the Institutional Review Board (IRB) at the Providence VA Medical Center and were conducted in accordance with the ethical standards from the 1964 Declaration of Helsinki.

### Measures

#### Type of Death.

Type of death was determined to be by suicide or another means. The cause of death in the MDR is determined based on the patient’s death certificate. We extracted data about cause of death from the MDR.

#### Covariates.

Covariates for the primary analysis included age, sex, race/ethnicity, Elixhauser comorbidity index, ejection fraction, number of emergency department visits in the year prior to admission, psychiatric and medical inpatient admissions, healthcare costs in the year prior, and length of stay for the last HF admission. These data were extracted from the VA electronic health record.

### Data Analysis

Baseline characteristics across the suicide versus nonsuicide related deaths included t-tests for continuous variables and *χ*^2^ tests for categorical variables. Due to the large sample size, standardized mean differences were calculated for all variables as a significant result may not be meaningful unless there is also an observed effect. Cohen’s taxonomy of 0.20, 0.50, and 0.80 for small, medium, and large effects were used to interpret the standardized mean differences.^23^

The primary analysis was predicting death by suicide within 1-year after a participant’s most recent heart failure admission using Cox proportional hazard models. In so doing, given research showing that suicide risk is particularly elevated in the 1-month, up to a year following an inpatient admission in other types of hospital stays (i.e., psychiatric inpatient admission), but ebbs and flows over that 1-year period, we chose to examine 30-day, 90-day and 1-year death outcomes. We also examined death up to 2 years to examine patterns of suicide death versus other forms of death in the year thereafter, for a total of four separate models (one per outcome). Of note, some deaths by suicide occurred more than 9 years after hospitalization. We chose a maximum timeframe of 2 years in our models because 1) we were interested in understanding how risk for death by suicide may be linked with a medical hospitalization event, and 2) to mimic the timeframes used in research examining suicide risk following other types of inpatient discharge (i.e., psychiatric inpatient stay). We ran both adjusted (models controlling for covariates described above) and unadjusted models, examining the assumption of proportional hazards in each model. Propensity score matching with 1:100 matching (i.e., for every one participant who died by suicide, we matched with 100 participants who died due to other causes) was used to balance the suicide versus nonsuicide related death groups to be consistent with best practices.^24,25^ We also conducted a sensitivity analysis using inverse probability of treatment weights (IPTWs) in addition to our main analyses. All analyses were conducted in SAS 9.4 (SAS Institute, Inc., Cary, NC).

## RESULTS

### Group Differences by Type of Death

Overall, there were 1097 suicides in our total original cohort of 745,491 Veterans with a primary admissions diagnosis of heart failure (inclusive of patients regardless of whether they lived or died). This equates to a rate of 147.2 deaths by suicide per 100,000 people before controlling for covariates. Sample characteristics, including differences by type of death, are shown in Table 1. Notable differences included the individuals who died by suicide being younger (Standardized mean difference [SMD] = 0.46), having fewer comorbidities (Standardized mean difference [SMD] = 0.28), being more likely to have a depression diagnosis (SMD = 0.26), being more likely to be White (SMD = 0.41), and having lower healthcare costs in the past year (SMD = −0.25).

### Time Until Death

Table 2 shows differences in the number of days from discharge and time to death. The individuals who died via suicide had a longer length of time between discharge and death on average (SMD = 0.18 Moreover, those who died by suicide were less likely to have died within 30-days (SMD = −0.13), 90-days (SMD = −0.21), 1-year (SMD = −0.20), and 2-years (SMD = −0.21) since discharge compared to individuals who died due to other reasons (see Table 2). The unadjusted models showed a negative association between time until death and type of death (i.e., suicide versus other causes). However, after using propensity score matching, the adjusted models suggested type of death was not associated with time until mortality at 30-days (Hazard Ratio [HR] = 1.01, 95% Confidence Interval [CI] 0.86, 1.18), 90-days (HR = 0.96, 95% CI 0.85, 1.08), 1-year (HR = 0.99, 95% CI 0.90, 1.08), or 2-years (HR = 1.00, 95% CI 0.92, 1.08) later. A sensitivity analysis using IPTWs showed no major changes in primary outcomes. Full reporting of the unadjusted, propensity score, and IPTW models is shown in Table 3.

### Conclusions

Although HF has been linked with increased risk of suicide, few studies have examined suicide risk in the months after discharge from a heart failure-related hospitalization. This study was the first to examine patient characteristics associated with suicide deaths in HF, and to examine whether the time to death after discharge from a medical inpatient stay differed amongst patients who died by suicide versus other causes. Overall, there were 147.2 deaths by suicide per 100,000 people before controlling for covariates, far exceeding typical rates of suicide in same-aged Veterans (about 35 per 100,000; 2024 National Veteran Suicide Prevention Annual Report), highlighting suicide as an important concern among patients with a recent heart failure-related hospitalization in particular. We also found that those who died by suicide were younger, had fewer medical comorbidities, more psychiatric comorbidities, and lower recent healthcare costs. Moreover, approximately half of patients who died by suicide died within a year of discharge from their most recent heart failure admission. Interestingly, although patients who died by suicide had a longer length of time between discharge and death on average compared to those who died by other causes, there was no difference in the time to death across death types after adjusting for indices of general medical health covariates. These results have important implications for efforts to prevent suicides among patients with HF.

Our results showed there were multiple, clinically meaningful differences between Veterans with a recent HF admission who died by suicide versus other causes. In particular, patients who died by suicide were younger (*M*_age_ 68.6 versus 73.7), and more likely to be White. This is consistent with reported suicide deaths within Veterans Health Administration^26^ and for older adults in the general population.^27^ Those who died by suicide did not differ by gender, which diverges from findings in civilian and Veteran samples, where men are more likely to die by suicide.^28,29^ However, our sample was comprised almost entirely of men (~98%), which likely precluded our ability to detect these effects. Notably, those who died by suicide were physically healthier – they had less severe HF presentations (i.e., were less likely to have a congestive heart failure diagnosis, less likely to have pulmonary circulatory or perivascular issues), and fewer medical comorbidities. They also had shorter acute inpatient lengths of stay and lower healthcare costs in the year prior to admission. However, those who died by suicide had greater psychiatric comorbidities (i.e., alcohol and drug use disorders, depression). These findings are consistent with prior findings that suicide decedents generally had fewer physical comorbidities but more psychiatric comorbidities than those who died by “natural causes”.^30^ Neuropsychiatric factors, in particular, were uniquely associated with suicide outcomes among older adult Veterans.^31^

We also examined the timing of death by suicide following discharge from a HF admission. The median survival time of patients who died by suicide was just over a year postdischarge. Among patients who died by suicide, we found that approximately 16% died within 1 month of discharge, 26% within 3 months, and 49.5% within a year of their most recent heart failure-related admission. While suicide risk is elevated in the year following discharge from psychiatric hospitalization,^32^ these findings suggest discharge from medical hospitalization may also represent a period of heightened risk for suicide among those with HF. That half of the suicides occurred within a year of discharge, with an additional 13.5% dying in the second year, and 37% more than 2 years after discharge may have important implications for efforts to reduce excess deaths among those with HF. Brief suicide prevention assessment and interventions have led to reductions in suicidal behavior following discharge from emergency departments in the form of safety planning with post-discharge phone contacts^33,34^ and with crisis response planning^35^ regardless of primary concern at admission. Our findings suggest targeted assessment and intervention may be effective in lengthening time to death following discharge from HF admission. Further research is needed to clarify factors associated with suicide risk following discharge from medical hospitalization.

Results of analyses examining the time to death as a function of suicide versus other causes of death were more surprising. Overall, we found that patients who died by suicide generally lived more than 150 days longer after being discharged from a heart failure-related hospitalization than patients who died from other causes. However, after adjusting for relevant covariates in proportional hazard models, type of death was not associated with time to mortality at 30-days, 90-days, 1-year, or 2-years following discharge. Therefore, after accounting for demographic covariates, medical comorbidities, and healthcare utilization metrics, differences between groups are ameliorated. This suggests that differences in time to death may be explained at least partially by demographic and medical comorbidities (i.e., depression) linked with suicide versus other forms of death.

There are several potential implications for these findings in the care of patients with HF. That suicide deaths occurred later than deaths by other causes suggests there may be clinical opportunities to consult or engage patients with mental health services in the year following hospital discharge. In particular, findings regarding psychiatric comorbidities suggest that outpatient medical providers might consider screening for comorbid factors like depression or alcohol/drug use disorders among patients with HF following hospitalization. Brief cognitive behavior therapy has been shown to reduce suicidal ideation among Veterans with chronic illness (including HF), and may have promise following hospitalization.^36^ Elements of the Zero Suicide Framework, an integrative systematic approach to suicide prevention across healthcare specialties, have shown preliminary feasibility in medical settings.^37,38^ Examination of coordinated Zero Suicide framework interventions may thus be warranted following hospitalization for HF. Brief interventions targeting modifiable factors uniquely associated with suicide among older adults including hopelessness and social disconnectedness may be particularly beneficial.^39-41^

Study findings must be considered in light of several limitations. First, due to the configuration of the present dataset, specific death types were not examined in the present study. Therefore, we are unable to make conclusions regarding specific causes of death in comparison to those who died by suicide. It is also possible that some deaths were misclassified on the death certificate (i.e., deaths classified as accidental overdoses/deaths versus suicides when intent could not be clearly inferred). Second, while this study sample had considerable geographic and racial/ethnic diversity, the vast majority of the study sample were male. Thus, findings may not generalize to women with recent hospitalization from HF. Due to use of electronic medical record data, we were unable to ascertain other proximal factors potentially contributing to suicide risk among patients with HF, including adjustment following hospitalization, quality of life, and life stressors. Moreover, we were unable to examine how attendance (or lack thereof) at other types of health care visits (i.e., outpatient psychiatric visits or therapy visits) may have contributed to the pattern of findings observed in our study. Future research should examine the potential impact of psychological and physical functioning, as well as other forms of treatment, on suicide-related behaviors among those recently hospitalized with HF. Finally, this study did not examine whether there were particular patient characteristics that differentiated individuals with a recent HF-related admission who died by suicide from those who lived; this is an important direction for future research.

Despite these limitations, the present study has several key strengths. This is a large, well-powered study, representing nearly 350,000 patients, allowing for detection of small effect sizes. Use of data from the Veterans Affairs Medical Centers across the United States allows for geographic and racial/ethnic diversity within the study sample. Further, the use of linked data sources allows for examination of factors related to death by suicide and other causes.

## Figures and Tables

**TABLE 1.** Sample Characteristics

Characteristic	Overall N = 348,840	Mortality Due to Suicide N= 1,097	Mortality Due Other Reason N= 347,743	Standardized Mean Difference	p-Value
Male, N (%)	341,680 (97.9%)	1,081 (98.5%)	340,599 (97.9%)	0.05	0.16
Age in years at most recent admission, M (SD)	73.7 (11.1)	68.6 (11.3)	73.7 (11.1)	−0.46	<0.001
Age group, N (%)				0.42	<0.001
18–64	80,149 (23.0%)	407 (37.1%)	79,742 (22.9%)	0.42	<0.001
65–74	94,088 (27.0%)	340 (31.0%)	93,748 (27.0%)		
75–84	110,489 (31.7%)	268 (24.4%)	110,221 (31.7%)		
85+	64,114 (18.4%)	82 (7.5%)	64,032 (18.4%)		
Race or ethnicity, N (%)					
White	255,889 (73.4%)	920 (83.9%)	254,969 (73.3%)	0.41	<0.001
Black or African American	54,943 (15.8%)	47 (4.3%)	54,896 (15.8%)		
Hispanic	32,933 (9.4%)	106 (9.7%)	32,827 (9.4%)		
Missing	3,798 (1.1%)	20 (1.8%)	3,778 (1.1%)		
Sum of Elixhauser comorbidity index, M (SD)	5.9 (3.0)	5.1 (2.8)	5.9 (3.0)	−0.28	<0.001
Congestive heart failure diagnosis, N (%)	236,010 (67.7%)	652 (59.4%)	235,358 (67.7%)	−0.17	<0.001
Valvular disease, N (%)	65,119 (18.7%)	166 (15.1%)	64,953 (18.7%)	−0.09	0.003
Pulmonary circulation disorders, N (%)	41,558 (11.9%)	94 (8.6%)	41,464 (11.9%)	−0.11	0.001
Peripheral vascular disorders, N (%)	94,000 (26.9%)	203 (18.5%)	93,797 (27.0%)	−0.20	<0.001
Paralysis, N (%)	7,487 (2.1%)	22 (2.0%)	7,465 (2.1%)	−0.01	0.74
Other neurological disorders, N (%)	34,549 (9.9%)	71 (6.5%)	34,478 (9.9%)	−0.13	<0.001
Chronic pulmonary disease, N (%)	172,695 (49.5%)	506 (46.1%)	172,189 (49.5%)	−0.07	0.02
Diabetes diagnosis, N (%)	173,965 (49.9%)	448 (40.8%)	173,517 (49.9%)	−0.18	<0.001
Hypertension diagnosis, N (%)	284,641 (81.6%)	857 (78.1%)	283,784 (81.6%)	−0.09	0.003
Arrythmia diagnosis, N (%)	176,398 (50.6%)	463 (42.2%)	175,935 (50.6%)	−0.17	<0.001
Hypothyroidism, N (%)	45,987 (13.2%)	109 (9.9%)	45,878 (13.2%)	−0.10	0.002
Renal failure diagnosis, N (%)	116,412 (33.4%)	211 (19.2%)	116,201 (33.4%)	−0.33	<0.001
Liver disease, N (%)	26,087 (7.5%)	71 (6.5%)	26,016 (7.5%)	−0.04	0.20
Ulcer diagnosis, N (%)	10,238 (2.9%)	26 (2.4%)	10,212 (2.9%)	−0.04	0.27
Metastatic cancer, N (%)	14,771 (4.2%)	16 (1.5%)	14,755 (4.2%)	−0.17	<0.001
Solid tumor without Metastasis, N (%)	67,766 (19.4%)	149 (13.6%)	67,617 (19.4%)	−0.16	<0.001
Rheumatoid arthritis, N (%)	11,911 (3.4%)	35 (3.2%)	11,876 (3.4%)	−0.01	0.68
Coagulopathy, N (%)	32,532 (9.3%)	66 (6.0%)	32,466 (9.3%)	−0.12	<0.001
Obesity diagnosis, N (%)	61,685 (17.7%)	231 (21.1%)	61,454 (17.7%)	0.09	0.003
Unintended weight loss, N (%)	36,196 (10.4%)	76 (6.9%)	36,120 (10.4%)	−0.12	<0.001
Fluid and electrolyte disorders, N (%)	115,392 (33.1%)	256 (23.3%)	115,136 (33.1%)	−0.22	<0.001
Blood loss anemia, N (%)	47,407 (13.6%)	106 (9.7%)	47,301 (13.6%)	−0.12	<0.001
Deficiency anemia, N (%)	8,611 (2.5%)	15 (1.4%)	8,596 (2.5%)	−0.08	0.02
Alcohol abuse, N (%)	26,768 (7.7%)	127 (11.6%)	26,641 (7.7%)	0.13	<0.001
Drug abuse, N (%)	16,914 (4.8%)	91 (8.3%)	16,823 (4.8%)	0.14	<0.001
Psychosis, N (%)	28,856 (8.3%)	66 (6.0%)	28,790 (8.3%)	−0.09	0.01
Depression, N (%)	97,612 (28.0%)	443 (40.4%)	97,169 (27.9%)	0.26	<0.001
Acute inpatient length of stay 1 year prior to most recent admission, M (SD)	7.9 (14.8)	4.9 (11.5)	7.9 (14.9)	−0.23	<0.001
Number of acute inpatient stays 1 year prior to most recent admission, M (SD)	1.3 (1.8)	1.0 (1.6)	1.3 (1.8)	−0.20	<0.001
Number of emergency room visits 1 year prior to most recent admission, M (SD)	1.9 (3.8)	1.7 (3.7)	1.9 (3.8)	−0.06	0.07
Ejection fraction value, M (SD)	41.6 (15.6)	42.7 (15.6)	41.6 (15.6)	0.07	0.06
Missing EF data, N (%)	114,167 (32.7%)	421 (38.4%)	113,746 (32.7%)		
Ejection fraction category					
0-40%	102,879 (29.5%)	286 (26.1%)	102,593 (29.5%)	0.15	0.001
40-50%	45,775 (13.1%)	137 (12.5%)	45,638 (13.1%)		
50+%	86,019 (24.7%)	253 (23.1%)	85,766 (24.7%)		
Missing	114,167 (32.7%)	421 (38.4%)	113,746 (32.7%)		
Total costs in past year, M (SD)	43,757.5 (73,233.4)	28,017.4 (50,399.8)	43,807.2 (73,288.8)	−0.25	<0.001

**TABLE 2.** Time Until Death by Reason for Death

Characteristic	Overall N = 348,840	Mortality Due to Suicide N = 1,097	Mortality Due Other Reason N = 347,743	Standardized Mean Difference	p-Value
Days from discharge until death, M (SD)	619.6 (914.1)	789.4 (1,002.8)	619.1 (913.8)	0.18	<0.001
Days from discharge until death, Mdn (IQR)	216.0 (43.0, 816.0)	374.0 (80.0, 1158.0)	215.0 (43.0, 815.0)		
Mortality within 30 days, N (%)	71,317 (20.4%)	170 (15.5%)	71,147 (20.5%)	−0.13	<0.001
Mortality within 90 days, N (%)	124,802 (35.8%)	288 (26.3%)	124,514 (35.8%)	−0.21	<0.001
Mortality within 1 year, N (%)	207,491 (59.5%)	544 (49.6%)	206,947 (59.5%)	−0.20	<0.001
Mortality within 2 years, N (%)	253,952 (72.8%)	694 (63.3%)	253,258 (72.8%)	−0.21	<0.001

**TABLE 3.** Cox Proportional Hazard Modeling Results

Model	Baseline Model		Propensity Score Matching		IPTW Weighted	
	Hazard Ratio	95% Confidence Interval	Hazard Ratio	95% Confidence Interval	Hazard Ratio	95% Confidence Interval
*30-day outcome*						
Unadjusted	0.73	0.63–0.85	1.01	0.86–1.18	1.05	0.92–1.20
Adjusted	0.94	0.81–1.09	1.01	0.86–1.18	1.13	0.99–1.30
*90-day outcome*						
Unadjusted	0.69	0.61–0.77	0.96	0.85–1.08	0.98	0.88–1.09
Adjusted	0.88	0.78–0.99	0.96	0.84–1.08	1.05	0.95–1.17
*1-year outcome*						
Unadjusted	0.74	0.68–0.80	0.98	0.90–1.07	1.01	0.93–1.10
Adjusted	0.93	0.86–0.99	0.99	0.90–1.08	1.09	1.00–1.18
*2-year outcome*						
Unadjusted	0.75	0.70–0.81	0.99	0.91–1.07	1.00	0.93–1.08
Adjusted	0.94	0.81–1.01	1.00	0.92–1.08	1.08	1.00–1.16

Note: Adjusted models include sex, age, race, comorbidity, EF category, acute hospitalization length of stay during the 1 year prior, and total cost 1 year prior, number of ER visit 1 year prior, and VA hospital as covariates. Unadjusted models only account for VA hospital. The reported effect is for death due to suicide.

## Data Availability

The data supporting the findings of this study are not publicly available due to privacy or ethical restrictions.

## Highlights

- What is the primary question addressed by this study?
  This study examined patient characteristics associated with suicide deaths in heart failure, and examined whether the time to death after discharge from a medical inpatient stay differed amongst patients who died by suicide versus other causes.
- What is the main finding of this study?
  Half of patients who died by suicide died within 1-year of a heart failure admission. Although patients who died by suicide had a longer length of time between discharge and death on average compared to those who died by other causes, there was no difference in the time to death across death types after adjusting for relevant covariates.
- What is the meaning of the finding?
  These findings suggest there may be missed opportunities for clinical intervention to reduce preventable forms of death among heart failure patients in the transition from medical hospitalization.

## DATA STATEMENT

The data has not been previously presented orally or by poster at scientific meetings.
